# Time matters: Transcriptomic insights into temporally regulated reproductive and physiological processes in the life cycle of salps

**DOI:** 10.1371/journal.pone.0326246

**Published:** 2025-06-20

**Authors:** Svenja J. Müller, Ilenia Urso, Sara Driscoll, Katharina Michael, Gabriele Sales, Cristiano de Pittà, Wiebke Wessels, Bettina Meyer

**Affiliations:** 1 Institute for Chemistry and Biology of the Marine Environment, Carl von Ossietzky University of Oldenburg, Oldenburg, Germany; 2 Scientific Division Polar Biological Oceanography, Alfred Wegener Institute Helmholtz Centre for Polar and Marine Research, Bremerhaven, Germany; 3 Department of Biology, University of Padova, Padova, Italy; 4 Helmholtz Institute for Functional Marine Biodiversity at the University of Oldenburg (HIFMB), Oldenburg, Germany; Laboratoire Arago, FRANCE

## Abstract

Despite the increasing importance of salps and the recognition of their role as important players in food webs and biogeochemical cycles, their life cycle characteristics and physiology remain mysterious. This uncertainty encourages oversimplifying modeling approaches, leading to inaccuracies that may affect population dynamics results. This lack of knowledge is critical, making it difficult to adequately assess their sensitivity to global warming and their impact on ecosystems if their abundance and distribution change with rising seawater temperatures. Therefore, we generated a *de novo* transcriptome of *Salpa fusiformis* to further investigate the physiological processes involved in the life cycle of salps. We examined differentially expressed genes between both reproductive forms, blastozooids and oozoids, and detected a general form-specific difference among *Salpa*. Furthermore, we identified mainly temporally driven processes (energy delivery, cell communication, spermatogenesis) by studying gene expression profiles of different developmental stages of blastozooids of *Salpa fusiformis*. A life cycle that physiologically prepares blastozooids during the potential reproductive period, regardless of their fertilization status, may favor rapid response to favorable conditions and formation of salp blooms. Understanding the processes involved will contribute to a better assessment of their sensitivity to environmental change and support the implementation of their role in ecosystem models. In addition, the generated transcriptome was used to select and validate a set of potential reference genes for future qPCR applications. This will facilitate molecular studies of tunicates generally and stimulate future physiological studies on salps.

## Introduction

Salps are pelagic tunicates found throughout the world`s ocean, with the exception of Arctic regions [1]. They are efficient filter-feeders [2], allowing them to exceed the total primary production of an area when they are abundant [3]. It is estimated that 74% of total primary production in the Mediterranean Sea may be removed by salps [4]. In this way they can alter the structure of the pelagic food web by displacing other grazers [1,5]. Salps are also known to rapidly ‘repack’ sinking faecal pellets, a process that can significantly affect biogeochemical cycles, particularly during massive occurrences known as ‘blooms’ [6–8].

The development of such salp blooming events is facilitated by the rapid reproduction of the asexual reproductive form (oozoid) by producing genetically identical clones of chains (blastozooids) through budding. Salps have a complex life cycle, with two generations alternating between an asexual (oozoid), and a sexual reproductive form (blastozooid). Blastozooids are born as females, and reproduce sexually when they reach maturity, developing a single embryo within their body. It is generally believed that blastozooids are sequentially hermaphroditic, producing testes to become males after the embryo is released. After their sexual transition, the male blastozooids fertilize younger female blastozooids [5,9,10]. The alternation between asexual and sexual reproduction therefore allows populations to grow while maintaining genetic variability [8,9,11].

The role of salps, particularly of the Southern Ocean salp *S. thompsoni*, in global pelagic ecosystems has been re-evaluated as their abundance and distribution appear to have changed, probably as a direct result of global warming [6,12,13]. However, it is questionable if *S. thompsoni* is able to maintain their populations in high Antarctic regions, as reproduction appeared to suffer from unfavorable temperatures and/or scarcity of food [14–16]. These effects appeared to be particularly relevant to the sexually reproducing blastozooids, as no negative effects on reproductive capacity of oozoids were observed [16]. More specifically, unfavorable environmental conditions led to an increased number of failed embryos, which may have been re-absorbed by their parents [15]. To properly understand future dynamics of the salp life cycle and to accurately incorporate salps into ecosystem models, we need to understand ongoing processes during development and reproduction, which is key to determining the most vulnerable stage under future climate change scenarios. Progress has been made in establishing models considering their life cycle under current and future climate change scenarios [17–20]. However, the unique reproductive strategy of salps in modelling approaches is often oversimplified and cohort analyses always assume a positive relationship between individual growth and development [21–23].

Considering this uncertainty, our aim was to identify processes involved in blastozooid development over time depending on the developmental stage, as sexual reproduction and therefore, the successful development of the embryo is hypothesized to be a crucial factor in the persistence of, e.g., *S. thompsoni* populations [16]. Given its morphological and developmental similarity to *S. thompsoni* [11,24], we therefore generated a *de novo* transcriptome of *S. fusiformis*, a widespread and dominant salp species during spring blooms in Mediterranean coastal waters [25,26]. As observed in other widely distributed gelatinous planktonic species [27,28], some degree of underlying genetic divergence—and potentially cryptic diversity —cannot be entirely ruled out in salps. However, such diversity has not been documented for *S. fusiformis* to date and remains beyond the scope of the present study.

Here, we aimed to provide deeper insights into the temporally regulated physiological processes underlying blastozooid development in this closely related species as well as its ecological implications. To be able to fully cover the reproductive cycle, focusing on the sexual reproducing blastozooids, we kept *S. fusiformis* in a newly developed kreisel tank system for several days during a spring bloom in 2021 [23]. This allowed us to maintain salps in controlled conditions and to sample at regular intervals. In addition, field samples of *S. fusiformis* from 2017 spring bloom were used to contribute to the generation of the transcriptome of *S. fusiformis*, which has already proven to be a powerful tool for studying the physiology of gelatinous organisms [29–31]. We identified potential form- (blastozooids and oozoids) and stage-specific (within blastozooids) differences in gene expression patterns of *S. fusiformis* during the reproductive cycle, providing new insights into the reproductive ecology of salps. In addition, several ‘housekeeping’ genes were identified that showed the greatest stability in the transcriptome across all reproductive forms and stages. The suitability of these candidates was validated by qPCR, providing a set of universal housekeeping genes and thus, a basis for future molecular studies. By identifying the processes involved in successful sexual reproduction on gene expression level, we can better assess the effect of changing environmental conditions on reproductive success and therefore, their ability to proliferate, e.g., in formerly non-salp-dominated areas as currently observed in the Southern Ocean [6,12]. In addition, insights into their development may help to more accurately include life cycle dynamics into ecosystem models in future studies.

## Results

### *De novo* transcriptome analysis of *S. fusiformis*

**Fig 1:**
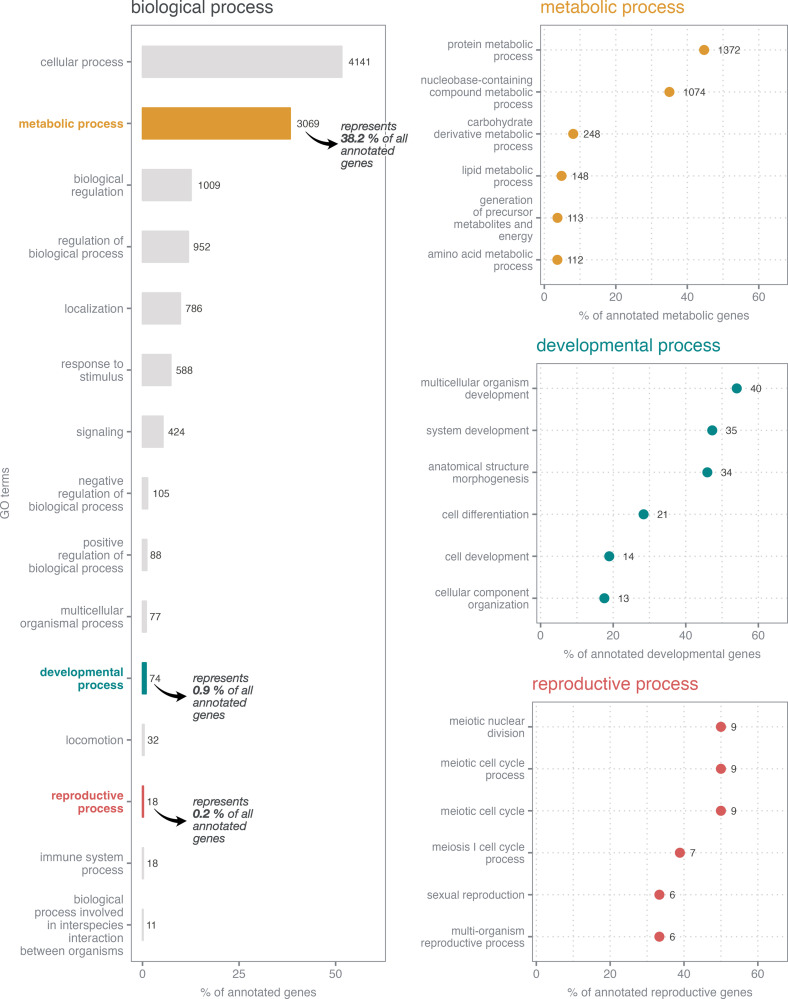
Gene ontology term distribution of annotated genes in the transcriptome of *S. fusiformis.* Numbers next to the bar/dot indicate the absolute number of genes assigned to each category, while the x- axis indicates the respective percentage. Classification of biological processes (left) of all annotated genes (8,028) in the reference transcriptome, summarized at second level, is shown. Only categories that cover > 10 genes are shown. In addition, six representative GO terms (of fourth hierarchical level) within metabolic (3,069 genes), developmental (74 genes) and reproductive processes (18 genes) are presented (right).

For the construction of the *de novo* transcriptome of *S. fusiformis*, samples were collected during spring bloom sampling campaigns in 2017 and 2021 at the Institut de la Mer de Villefranche (France) (S1 Table in S1 File). The assembly of *S. fusiformis* contained 126,536 transcripts and a total of 80,186 genes with a median contig length of 655 bp and a N50 value of 2,394 bp. GC content of the transcriptome was 38.8%. About 87% of all reads were mapped back to the transcriptome. A eukaryotic database (eukaryota_odb10) was used as a reference, and BUSCO suggested an almost complete *de novo* assembly containing 95.3% of essential genes [(single-copy: 67.1%; duplicated: 28.2%), fragmented: 2.7%, missing: 2.0%]. Overall, by cross-referencing the results obtained from the searches against the different reference databases, a total of 84,285 sequences were assigned, representing 66.61% of the total number of reconstructed transcripts. Results from the functional annotation showed that 35.8% (45,319 transcripts) matched at least one protein from the NR database, while a search against NT nucleotide sequences resulted in 8.7% (11,040 transcripts) of matches. About 35.4% (44,770 transcripts) were annotated by TREMBL protein sequences. InterproScan analysis showed an annotation success of 61.4% (77,706 transcripts) and a total of 11,268 genes (14.1%) were associated with known and predicted proteins of which 8,028 (10.0%) were annotated with gene ontology (GO) terms. Most processes within biological processes (BP, 10.0%) were related to cellular (n = 4,141, 51.6%) and metabolic genes (n = 3,069, 38.2%), while genes involved in developmental (n = 74, 0.9%) and reproductive process (n = 18, 0.2%) were less represented (Fig 1). Metabolic genes were assigned to 182 GO terms at the fourth hierarchical level, with 44.7% (1,372 genes) of GO terms categorized as protein metabolic process. In contrast, only 4.8% (148 genes) and 8.1% (248 genes) belonged to lipid and carbohydrate metabolic processes, respectively. Among developmental processes, most genes were assigned to multicellular organism development (40 genes, 54.1%), system development (35 genes, 47.3%) and anatomical structure morphogenesis (34 genes, 46.0%) at the fourth hierarchical level (total number of GO terms n = 72). Within the GO term reproduction, most genes were related to meiotic cell cycle (9 genes, 50.0%), sexual reproduction (6 genes, 33.3%) and multi-organism reproductive process (6 genes, 33.3%), among others (total number of GO terms n = 35).

### Differential gene expression and functional analysis

#### Analysis of form effect (blastozooids vs. oozoids) in *S. fusiformis.*

**Fig 2:**
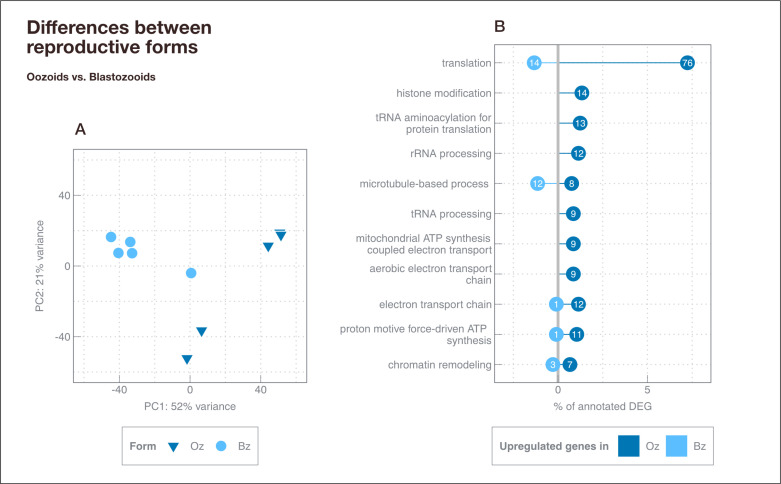
Differentially expressed genes between blastozooids (Bz) and oozoids (Oz) from campaign (1). (A) PCA shows variance-stabilized gene expression data. Different symbol color and shape indicate different forms (dark blue triangle = oozoids (Oz), light blue circle = blastozooid (Bz)). The explained variance [%] for PC1 and PC2 is given. (B) Statistically significant Gene Ontology terms (p < 0.05) for differentially expressed genes (DEG) between different reproductive forms are shown. Numbers in circles indicate the absolute number of genes assigned to each term, while the x-axis indicates the respective percentage of all annotated DEG (1,050 DEG, 26.8% annotation). Only statistically significant terms covering ≥ 8 DEGs are shown. See S2 Table in S1 File for a full list of all enriched GO terms within BP.

Field samples from campaign 1 (see chapter *Material and Methods – Field sampling and experimental design*) were used to compare gene expression profiles between blastozooids (n = 5) and oozoids (n = 5) in *S. fusiformis* (S1 Table in S1 File). An embryo was present in all blastozooids before being removed. However, we could not obtain information on the exact development stage of blastozooids and oozoids. After filtering for a minimum of ten total counts per gene in samples from campaign 1 (n = 10), 46,838 genes were retained. Principal component analysis (PCA) showed that most of the variance could be explained by the two reproductive forms (PC1: 52%, blastozooids vs oozoids; Fig 2A). Differential gene expression (DGE) analysis revealed 3,913 (LFCT = 0, BH adjusted p-value < 0.05) differentially expressed genes (DEG) between blastozooids (n = 5) and oozoids (n = 5), of which 26.8% (1,050 genes) were annotated. About 66.7% of DEG were upregulated in blastozooids compared to oozoids. GO term enrichment analysis revealed 19 terms (p < 0.05) within BP (Fig 2B, S2 Table in S1 File). Most genes (n = 90) were assigned to translation. 84.4% (n = 76) of these genes were upregulated in oozoids, with the majority of them encoding for ribosomal proteins (large and small subunits), different translation initiation and elongation factors (Translation elongation factor EF1B, Translational (tr)-type GTP-binding domain) and aminoacyl-tRNA synthetases (Lysyl tRNA synthetase, class II, C-terminal, Serine-tRNA ligase, type1, Glycyl tRNA synthetase, Tyrosine-tRNA ligase). In addition, genes involved in energy metabolism (e.g., electron transport chain, mitochondrial ATP synthesis coupled proton transport) were found to be upregulated in oozoids, including different subunits of cytochrome c oxidase, NADH dehydrogenase [ubiquinone], ATP synthase (F0 complex, subunit C) and ATPase (F1/V1/A1 complex; OSCP/delta subunit). In contrast, about 60% of genes related to microtubule-based process (ankyrin repeat, tubulin, kinesin motor domain, dynein heavy/light chain) were found to be upregulated in blastozooids compared to oozoids (Fig 2B).

#### Analysis of development and fertilization stages in blastozooids of *S. fusiformis.*

**Fig 3:**
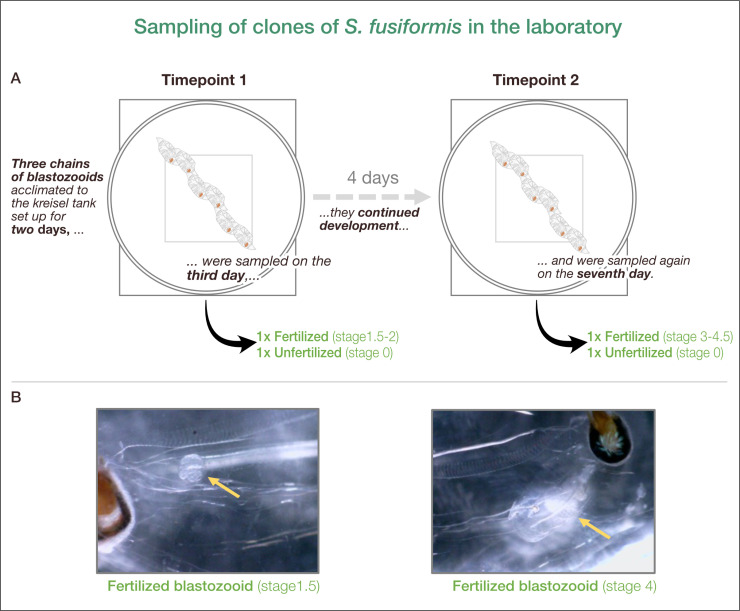
Schematic representation of experimental design of salps obtained during sampling campaign (2). (A) During each sampling timepoint one fertilized and one unfertilized blastozooid of each chain (n = 3) was sampled from a kreisel tank. (B) Exemplary pictures of fertilized blastozooids taken during the sampling campaign (2) at the Institut de la Mer de Villefranche (France) in 2021. Blastozooids of stage 1.5 at timepoint 1 (left) and stage 4 at timepoint 2 (right) are shown (staged according to Foxton et al. (1966)). Yellow arrow highlights the embryo. Sperm channel started to become visible on the salp nucleus (right).

Samples (n = 12) from campaign 2 (see chapter *Material and Methods – Field sampling and experimental design*) were used to compare different developmental stages within blastozooids. Sampling was performed after salps had been maintained for 3 (TP1) and 7 (TP2) days in the kreisel tank system (Fig 3, S1 Table in S1 File). At each sampling timepoint, one fertilized and one unfertilized blastozooid of each chain (n = 3) was sampled, allowing comparison of different developmental stages within the same chains (clones). Blastozooids were staged based on embryo development according to Foxton et al. (1966). Unfertilized blastozooids showed no presence of a developing embryo at TP1 (n = 3) and TP2 (n = 3). In contrast, fertilized blastozooids developed from stage 1.5−2 (n = 3) at TP1 to stage 3–4.5 (n = 3) at TP2 with 0.56 *± *0.17 (mean *± *SD) stage transitions day ^−1^ and a developmental rate of 1.91 *± *0.66 (mean *± *SD) days for performing one stage transition (S1 Table in S1 File). At TP1, fertilized blastozooids were freshly fertilized, with early signs of embryo development. At TP2, the embryos (~ 8 mm in total length) showed more advanced structures, including a visible placenta, eleoblast and embryo, with the embryo attached to the mother blastozooids and muscle structures clearly visible. We also observed that sperm channels became visible on the nucleus of the blastozooids at TP2 regardless of fertilization status (Fig 3).

**Fig 4:**
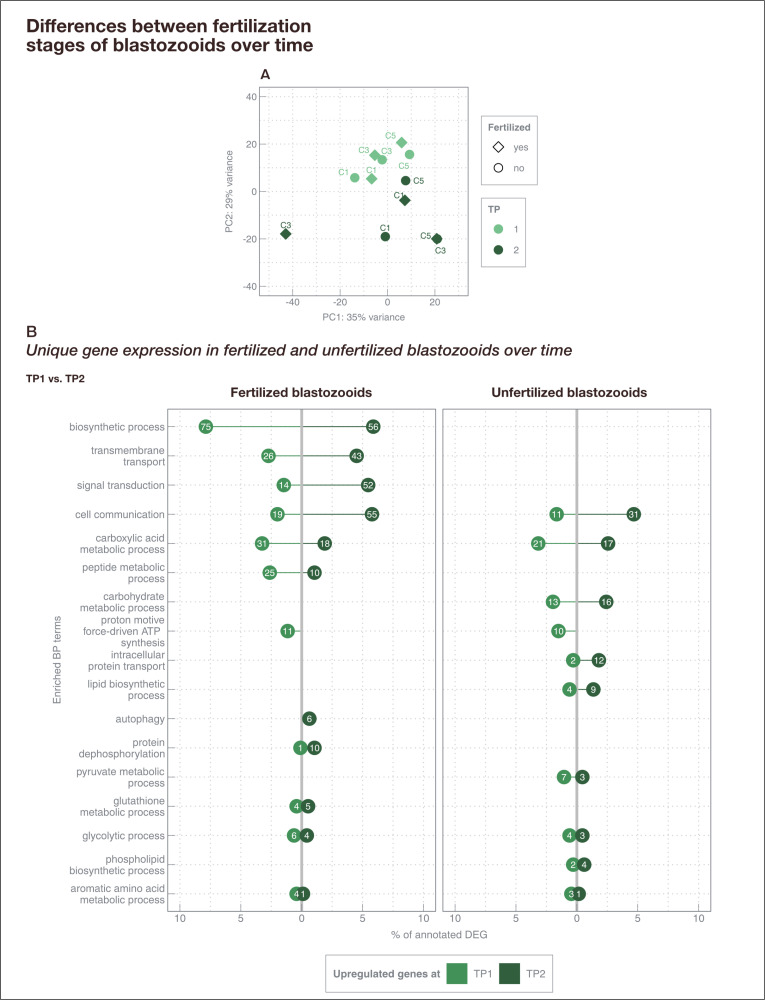
Differentially expressed genes between different fertilization stages of blastozooids over time from campaign (2). **(A)** PCA shows variance-stabilized gene expression data of 12 *S. fusiformis* samples from the kreisel tank system in spring 2021. Different symbol colors and shapes indicate different fertilization stages (diamond = fertilized, circle = unfertilized) and timepoints (light green = TP1, dark green = TP2). Identical point labels indicate individuals from the same chain (n = 3), i.e., clones. The explained variance [%] for PC1 and PC2 is also given. **(B)** Gene ontology terms (p < 0.05) enriched among differentially expressed genes between TP1 and TP2 in fertilized and unfertilized blastozooids. Numbers in circles indicate the absolute number of genes assigned to each GO term, while the x-axis indicates the percentage of all annotated DEGs within the respective comparison (fertilized: 1,789 DEG, 53.3% annotation; unfertilized: 1,427 DEG, 46.5% annotation).

After normalization and filtering (> ten counts per gene) of the count matrix obtained from RNA-Seq, 32,068 genes were retained for downstream analysis. Principal component analysis revealed, that samples rather clustered together by sampling timepoints (TP1, TP2) than by their fertilization status (fertilized, unfertilized; Fig 4A).

#### Analysis of gene expression depending on fertilization status (fertilized vs. unfertilized).

At the beginning of the experiment (TP1), only 16 genes (11 with annotation, 68.8%) were differentially expressed between fertilized and unfertilized blastozooids, increasing to 123 (56 with annotation, 45.5%) differentially expressed genes over the course of development (TP2) (comparison (a); *see Materials and Methods*). GO enrichment revealed six biological processes related to carboxylic acid metabolic process with 3 genes being upregulated in unfertilized blastozooids at TP1 (S3 Table in S1 File). Within biological processes 8 terms were found to be enriched (p < 0.05) at TP2 (S4 Table in S1 File). Most genes (n = 6) were assigned to carbohydrate derivative metabolic processes and generation of precursor metabolites and energy, of which most (~ 90%) genes were upregulated in unfertilized blastozooids.

#### Analysis of gene expression over time (TP1 vs. TP2).

Due to a pronounced temporal effect in gene expression signatures of samples from campaign 2, we focused in the following on temporally (TP1 *vs.* TP2) regulated gene expression patterns that are (1) unique to fertilized as well as unfertilized blastozooids, in order to detect processes involved in the successful development of the embryo and to identify potential additional load by the embryo on the maternal blastozooid compared to unfertilized blastozooids. Further, we investigated the general processes involved over time (2) independent of fertilization status.

#### (1) Unique gene expression patterns in fertilized and unfertilized blastozooids over time (TP1 *vs.* TP2):

Within fertilized blastozooids of *S. fusiformis,* analysis of differences in gene expression between timepoints (TP1 vs TP2) (comparison (b); *see Materials and Methods*) resulted in 1,789 DEG (953 with annotation, 53.3%). GO enrichment analysis revealed 25 significant terms (p < 0.05) within biological processes (S5 Table in S1 File), dominated by cellular and metabolic processes (n = 16) (Fig 4B). Most genes (n = 131, 13.8%) differentially expressed between TP1 and TP2 in fertilized blastozooids could be related to biosynthetic processes (e.g., glycogen synthase, ribosomal proteins, elongation factor 1B, serine hydroxymethyltransferase, ATP synthase coupling factor 6, Ubiquitin domain, Zinc finger, nuclear hormone receptor-type). In addition, ~ 14.7% (140 DEGs) of annotated genes were related to cell communication, transmembrane transport and signal transduction. Moreover, six genes were assigned to autophagy (Autophagy-related protein) and were found to be upregulated at TP2.

In unfertilized blastozooids, gene expression analysis between timepoints (TP1 vs TP2) (comparison (c); *see Materials and Methods*) resulted in 1,427 DEGs (663 with annotation, 46.5%). GO enrichment analysis revealed 31 enriched biological processes (S6 Table in S1 File), of which 16 terms covered cellular and metabolic processes (Fig 4B). As in fertilized blastozooids, the major process was cell communication, but the number of DEGs involved was lower. In addition, DEGs between timepoints were related to lipid/phospholipid biosynthetic process (13 DEGs, e.g., Malonyl-CoA decarboxylase, 3-oxo-5-alpha-steroid 4-dehydrogenase) and carbohydrate metabolic process (29 DEGs, e.g., Fructose-bisphosphate aldolase).

#### (2) Gene expression signatures in blastozooids between timepoints (TP1 vs TP2) independent of fertilization status:

**Fig 5:**
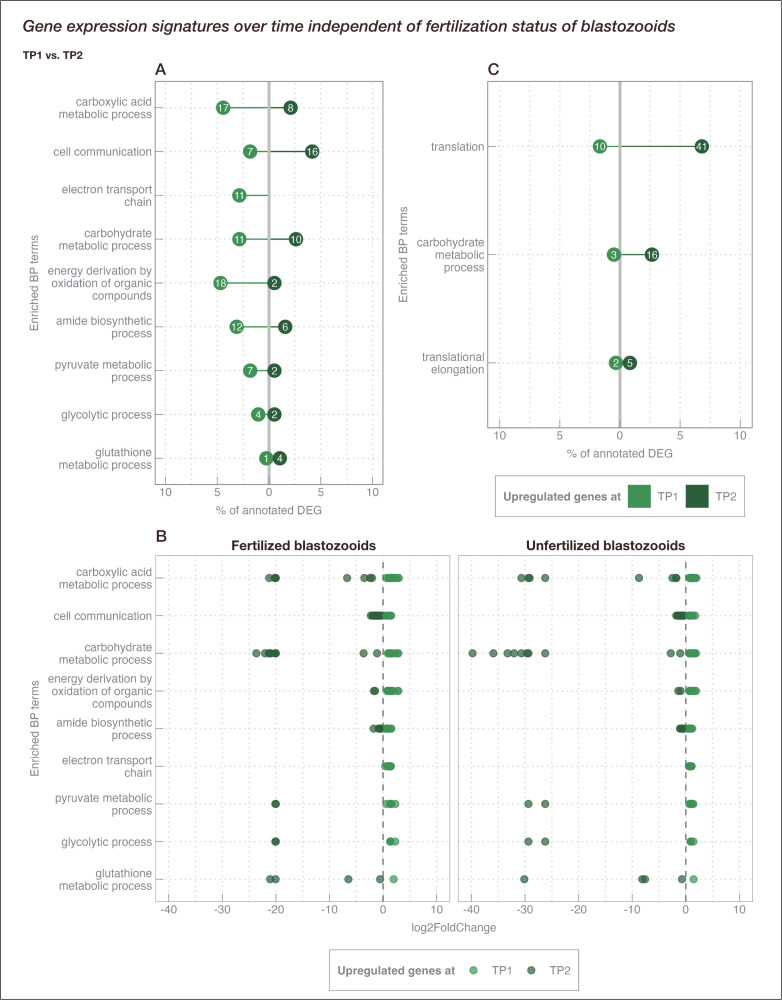
List of enriched Gene ontology terms (p < 0.05) in common (fertilized and unfertilized) differentially expressed genes between TP1 and TP2. **(A)** Genes that were found to be differentially expressed in fertilized and unfertilized blastozooids. **(B)** log2Foldchange of genes that were found to be differentially expressed in fertilized and unfertilized blastozooids (equal to (A)). Each data point represents a gene. The dashed vertical line marks the putative threshold for the upregulation during TP1 and TP2, respectively. Positive values indicate upregulation at TP1 while negative values show upregulation at TP2. **(C)** Comparison between TP1 and TP2 independently of the fertilization status (overall time effect). Numbers in circles indicate the absolute number of genes assigned to each term, while the x-axis indicates the percentage of all annotated DEG within the respective comparison (A: 721 DEG, 53.26% annotation; B: 1,802 DEG, 33.41% annotation).

In order to identify processes between timepoints and independent of fertilization status of blastozooids, genes identified in both, in fertilized and unfertilized blastozooids (see above), as well as DEGs over time, independent of fertilization (main effect time) were analyzed. Over time (TP1 *vs.* TP2), a total of 721 genes (384 with annotation, 53.3%) were found to be differentially expressed in both fertilized and unfertilized blastozooids (comparison (d); *see Materials and Methods*). Within biological processes 17 terms were found to be enriched (p < 0.05) (S7 Table in S1 File), 12 of which could be assigned to cellular and metabolic related terms (Fig 5A). In blastozooids of both states (fertilized and unfertilized), differential gene expression patterns identified between timepoints mainly involved cellular processes such as cell communication and metabolic processes such as carbohydrate, carboxylic acid metabolism, as well as energy-providing processes such as electron transport chain and pyruvate metabolic processes. DEGs involved in these processes were the most differentially expressed between timepoints compared to other processes involved, with upregulation during TP2 compared to TP1 (Fig 5B). This mainly involved carbohydrate breakdown processes, as the DEGs were glycolytic pathway components (enolase, Fructose-bisphosphate aldolase, Phosphoglycerate kinase, Glyceraldehyde-3-phosphate dehydrogenase, pyruvate kinase). Further DEGs of carboxylic acid metabolic processes were involved in beta-oxidation (Acyl-CoA dehydrogenase), and in the citric acid cycle (Lactate/malate dehydrogenase, Fumarat lyase). Expression magnitude (i.e., log2FoldChange) of genes annotated to these processes was similar in fertilized and unfertilized blastozooids (Fig 5B).

1,802 genes (602 with annotation, 33.4%) were differentially expressed over time independent of fertilization status (comparison (e); *see Materials and Methods*). GO enrichment analysis revealed 9 terms (p < 0.05) within biological processes (S8 Table in S1 File). The majority of DEGs were related to translation (n = 51) and translational elongation (n = 7), while ~80% of the genes were upregulated at TP2 compared to TP1 (Fig 5C). Further, a keyword search revealed that genes related to spermatogenesis (spermatogenesis-associated protein 48-like, spermatogenesis-associated protein 17-like) were differentially expressed between timepoints independent of fertilization status.

### RT-qPCR validation

**Fig 6:**
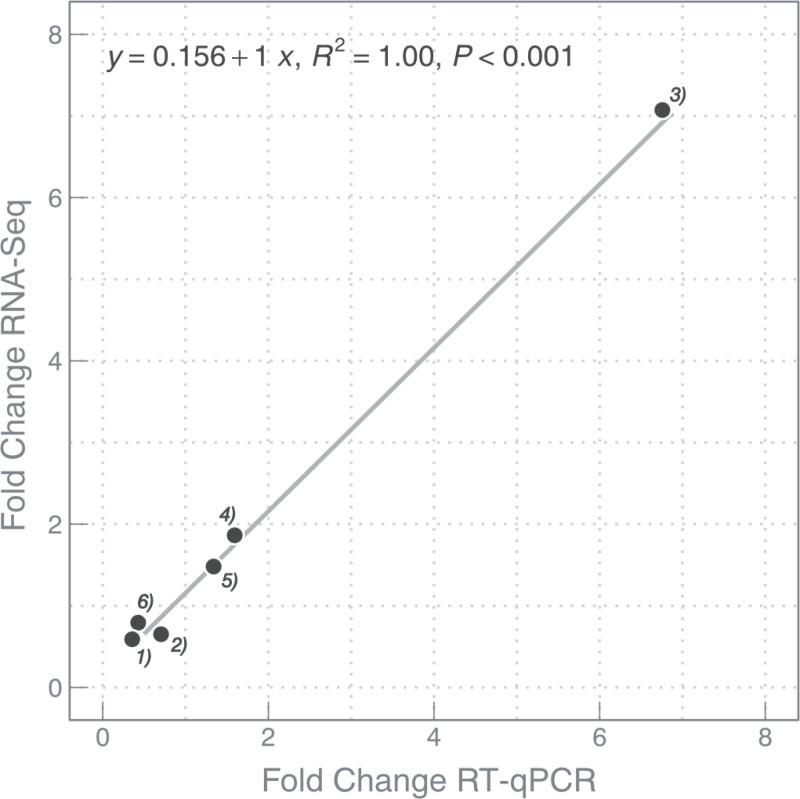
Validation of RNA-seq results using RT-qPCR. Each data point represents the fold change (2^-ΔΔCt^) of a gene selected among the enriched processes based on different contrasts (see *Material and Methods*). ^1)^*atpsyn*: blastozooids *vs*. oozoids, ^2)^*auto*: TP1 *vs.* TP2 (within fertilized blastozooids), ^3)^*tyramino*: TP1 *vs.* TP2 (within fertilized blastozooids), ^4)^*sperm48:* TP1 *vs.* TP2 (within fertilized blastozooids), ^5)^*sperm48:* TP1 *vs.* TP2 (within unfertilized blastozooids), ^6)^*cdan:* TP1 *vs.* TP2 (within unfertilized blastozooids).

In order to validate the results obtained by RNA-seq gene expression analysis and the selection of the respective reference genes for normalization, RT-qPCR was conducted for 5 representative genes (*atpsyn, auto, cdan, sperm48 and tyramino*). For selection and stability analysis of reference gene candidates see the supplemental text (S1 Text). Our results showed a highly significant positive correlation (p < 0.001, *R*^*2*^ = 1.00) between both methods, confirming the accuracy of the differential gene expression patterns identified here and also our selection of reference genes (Fig 6, S1 Text).

## Discussion

The Southern Ocean salp species, *S. thompsoni*, has gained increasing attention in recent years, as they have been observed to invade high Antarctic regions, which is likely to have drastic effects on the biogeochemical cycle due to its large grazing impact and rapidly sinking faecal pellets [7,12,32,33]. While the recognition of their ecological importance is increasing, profound knowledge of salp life cycle traits and physiology are still enigmatic, making it difficult to assess their ability to respond to ongoing environmental changes and, consequently, to predict future impacts on the ecosystem in a potentially salp-dominated zooplankton community. In order to improve our understanding of the processes during the development and reproduction of salps, we generated a *de novo* transcriptome of *S. fusiformis*, a close relative of *S. thompsoni,* in terms of body structure and developmental stages. As sexual reproduction in particular may be affected by unfavorable temperature and nutrient conditions and is also considered to be an important factor in the persistence of salp populations [15,16], we focused on the identification and characterization of processes involved in the development of blastozooids, during fertilization and embryo development as well as in the absence of fertilization. We therefore analyzed gene expression profiles between (1) both reproductive forms, blastozooids and oozoids, and (2) different developmental and fertilization stages of blastozooids over time.

### Functional annotation of the *de novo* transcriptome of *S. fusiformis*

We created a nearly complete transcriptome (95.3%) for *S. fusiformis* with high annotation success using various databases (NR, NT, TREMBL and InterproScan). However, the InterproScan annotation was the most successful, with 77,706 (61.4%) of 126,536 transcripts annotated. Among these, 11,268 genes (14.1%) were associated with known and predicted proteins. Compared to the *de novo* transcriptomes of the Southern Ocean salp *S. thompsoni,* generated by Batta-Lona *et al.* (2017) (18%) [30] and Müller *et al.* (2022) (37%) [29], our annotation success was lower. However, this can be explained by an additional step within the analysis to reduce redundancy by cross-checking transcript sequences and removing all sequences already represented by more than 90% in a longer transcript. In addition, the *de novo* transcriptome generated by Müller *et al.* (2022) [29] based on field samples and sequence data from Batta-Lona *et al.* (2017) [30], which led to a higher total number and coverage of sequences and likely, to a higher annotation rate.

GO term distribution analysis of the transcriptome of *S. fusiformis* revealed that most abundant terms were “cellular process” and “metabolic process” within biological process (BP) (Fig 1). While genes related to protein metabolism were most abundant (44.7%), the proportion of genes related to lipid (4.8%) and carbohydrate (8.1%) metabolic processes was low. This finding is consistent with the transcriptome analysis of the closely related species *S. thompsoni* [29]. A higher representation of genes belonging to protein metabolic processes may be in line with higher protein content of salps (10% of dry weight) [34].

### Gene expression patterns between reproductive forms suggest general differences in ribosomal capacities and energy metabolism among *Salpa*

Gene expression patterns differed between blastozooids and oozoids collected in the field in spring 2017 (Fig 2A), suggesting a general form-specific gene expression pattern in *S. fusiformis.* Most genes were found to be involved in the biological process translation, with the majority of genes upregulated in oozoids compared to blastozooids (Fig 2B). A higher translational activity in oozoids may be related to generally higher growth rates in oozoids compared to blastozooids [23,35]. However, only a few studies compared metabolic rates (e.g., oxygen consumption, ammonia excretion) between the two reproductive forms, and most of them only found none or non-significant differences [36,37]. Gene expression analysis in the Southern Ocean salp *S. thompsoni* revealed an upregulation of genes encoding for ribosomal proteins in oozoids compared to blastozooids between seasons and regions. However, it remained unclear whether the observed differences in translational capacity between the two forms were unique to the cold and unproductive environmental conditions or whether they also represented a general form effect [29,38]. Together with our results here we suggest that a higher investment in ribosomal capacity in oozoids compared to blastozooids, at least on transcriptional level, seems to be a general salp-specific trend in several species (*S. thompsoni, S. fusiformis*), possibly influenced by environmental conditions in addition. Also, differences in the expression of genes related to energy metabolism (electron transport chain, mitochondrial ATP synthesis coupled proton transport) were found between both reproductive forms of *S. fusiformis*, which may indicate different energy demands in oozoids compared to blastozooids. Since translation is an energy-consuming process, the upregulation of ribosomal capacity is consistent with a higher energy demand in oozoids compared to blastozooids [29,39]. Both reproductive forms differ substantially in morphology, which may explain the gene expression patterns observed here. The shape and number of muscle bands differ in size and arrangement between oozoids and blastozooids, resulting in different locomotory behavior [11]. In particular, blastozooids have fewer muscle bands, the musculature is weaker. They have also a different arrangement of dorsal eye and brain [40] and different reproductive structures than oozoids. While oozoids reproduce through stolon strobilization, blastozooids reproduce sexually and form an embryo and testis [11].

However, we cannot rule out the possibility that the trend observed here may be unique to spring bloom period. All samples used here were exclusively collected during a salp bloom in spring 2017. One of the main drivers for bloom formation is thought to be the rapid population growth through asexual reproduction by oozoids. Under favorable conditions, such as after a spring phytoplankton bloom, oozoids of *Thalia democratica* have shown to grow faster and form shorter blastozooid chains that mature more successfully which results in a larger bloom [41]. A higher investment in translational capacity and higher energy demand in *S. fusiformis* may therefore be reasonable in oozoids compared to blastozooids during a developing spring bloom (peak of salps at Point B: 49.03 individuals/m^3^ on 12.04.17 [42]). Interestingly, processes related to ribosome biogenesis, ATP catabolic processes and microtubule-based processes were also involved in the blastogenetic cycle (asexual reproduction) in the colonial ascidian *Botryllus schlosseri* [43]. Therefore, these processes seem to not only play a role in the (asexual) developmental cycle of the class *Ascidiacea* but may also be part of the underlying form-specific differences in gene expression in salps (*Thaliacea)* and may therefore be an integral component in the development of tunicates in general.

### Gene expression patterns between different fertilization and developmental stages of blastozooids point to a temporally regulated life cycle in salps

Low temperatures and unfavorable nutritional conditions are believed to affect sexual reproduction in salps, which is considered an important factor in the persistence of salp populations [15,16]. To better understand the processes occurring during sexual reproduction in blastozooids, gene expression patterns of blastozooids sampled after 3 (TP1) and 7 (TP2) days of maintenance in kreisel tanks [23] were obtained. During both samplings, one fertilized and one unfertilized blastozooid of each chain (n = 3) were sampled (Fig 3). While unfertilized blastozooids did not change their reproductive stage between both timepoints (stage 0), fertilized blastozooids developed within 4 days from stage 1.5–2 (TP1) to stage 3–4.5 (TP2). The PCA suggests that the difference in gene expression between the samples is rather driven by time than by the state of fertilization (Fig 4A). Therefore, we examined (1) the effects of successful embryonic development (from stage 1.5–2 to stage 3.5–4) on parental gene expression between TP1 and TP2 and compared them to unfertilized samples (stage 0), and (2) the differences in gene expression between both timepoints, regardless of fertilization status.

#### Biosynthesis and cell communication in fertilized blastozooids: insights into successful embryo development.

The majority of genes differentially expressed between both sampling timepoints during successful embryo development in blastozooids were related to biosynthetic processes, a process known to form more complex substances/structures while energy is consumed (Fig 4B). Specifically, these were mainly genes encoding for different subunits of ribosomal proteins. *S. fusiformis* belongs to the group of thecogene salps, which means that the embryo breaks through the follicular sac as it grows and enters the cloacal cavity of the mother. A circular fold of cloacal epithelium then grows around the embryo to cover it [11]. Therefore, the creation of complex structures for the development of an embryo, or perhaps some structural changes within the blastozooid itself, is reasonable while the embryo develops. This is consistent with the finding that no enrichment of comparable biosynthetic processes was found in unfertilized blastozooids between the two sampling timepoints. Signal transduction and cell communication seemed to play an important role over time (TP1 *vs.* TP2), particularly in fertilized blastozooids. These processes are often linked to extensive cellular and metabolic processes, such as ribosome biogenesis [44]. Therefore, the increased gene expression of cell communication between timepoints aligns with the biosynthetic processes involved in the temporal regulation in fertilized blastozooids. Furthermore, autophagy was found to be enriched in fertilized blastozooids. Autophagy can be associated with cellular stress and is indicative of the remodeling of intracellular structure for cell differentiation, which has also been shown to play a role in the development of the ascidian *Ciona intestinalis* [45]. Embryo growth also depends on a continuous supply of nutrients from the parental blastozooid [15], even when the embryo is fully developed and can filter its own food. The embryo is located behind the maternal filter and can only filter water already filtered by the parental blastozooid [11]. Therefore, the upregulation of autophagy-related processes at TP2 could be explained by an increasing dependence of the growing embryo on nutrient supply by the parental blastozooid.

#### Temporal regulation of energy production and reproductive readiness in blastozooids independent of fertilization.

**Fig 7:**
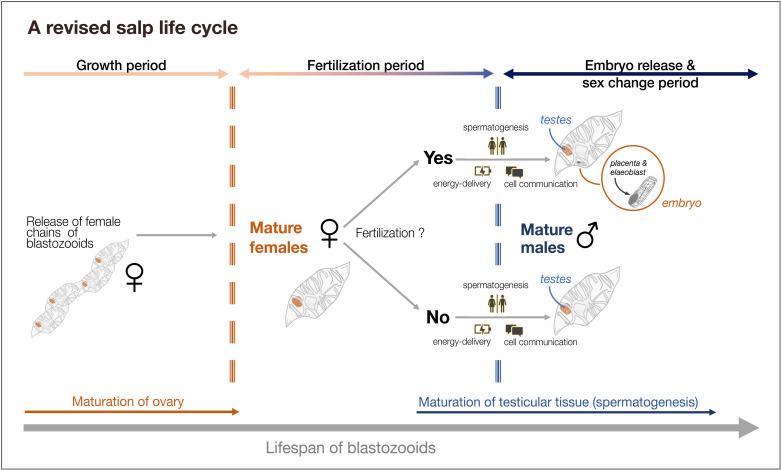
Schematic representation of the life cycle of salps. Salps are released as young female chains of blastozooids and grow until their ovaries mature. At a certain point, regardless of fertilization, salps begin to develop testicular tissue through primarily time-regulated physiological processes. A temporally regulated life cycle that physiologically prepares blastozooids similarly during the potential reproductive period, regardless of their fertilization status, may favor rapid response to favorable conditions and formation of salp blooms.

The majority of genes that were differentially expressed between timepoints independent of fertilization status (i.e., shared genes) were mainly related to cell communication and energy delivering processes (e.g., electron transport chain, energy derivation by oxidation of organic compounds) (Fig 5A). In addition, DEGs assigned to carbohydrate (glycolytic) processes (e.g., carbohydrate sulfotransferase, enolase) and pyruvate metabolic process (e.g., aldolase types, pyruvate kinase) were found to be enriched. Thus, energy producing processes seem to play an important role in the development of both fertilized and unfertilized blastozooids over time. Interestingly, no difference in the magnitude of the expression of genes related to energy-producing processes could be detected between fertilized and unfertilized blastozooids (Fig 5B). This could be because the selected genes are not directly involved in embryo maintenance or the embryos were still too small (~ 8 mm in total length) to impose significant energy costs on the fertilized mother blastozooid. Alternatively, blastozooids may be similarly physiologically prepared during the potential reproductive period, regardless of their fertilization status. This would allow unfertilized individuals to immediately cover potential energy surpluses resulting from a fertilization event. A life cycle with more precise temporal regulation, aligning blastozooids with the potential reproductive period regardless of prior fertilization, may promote rapid responses to favorable conditions and formation of salp blooms (Fig 7). An energy demand independent of fertilization status is also in line with growth rates observed for *S. fusiformis* in kreisel tanks, as they were not related to fertilization status [23]. Protein synthesis (translation) as the main process involved over time independent of fertilization status further underpins the continuous development and growth of blastozooids and the increased energy demand, regardless of fertilization (Fig 5C).

So far, salps were suggested of being sequential hermaphroditic, meaning that fertilization and the development of the embryo are prerequisites for the development of the testes to prevent self-fertilization [11]. However, we found spermatogenesis related genes were differentially expressed between timepoints regardless of the fertilization status. This is in line with recent findings of overlapping expression of male and female genes in blastozooid embryos of *S. thompsoni* [46] and morphological descriptions of *S. thompsoni* [47] suggesting an alternative hypothesis about hermaphroditism in salps. In our previous study, most of the blastozooids of *S. fusiformis* showed male characteristics (testis development) during TP2, even though they were unfertilized [23]. Along with the gene expression results found here, this suggests that testis development is not depending on previous fertilization but rather is developed time-controlled and occurs from a certain point onwards (Fig 7). This is also consistent with the finding that individuals of the same chains shed sperms together in time [48], further supporting our hypothesis that key physiological processes in the life cycle of salps are temporally regulated independent of their fertilization status. This would be in particular advantageous for the emergence of a bloom of *S. thompsoni*, especially in spring. While it is speculative whether (male) aggregates overwinter [29], it may be possible that the first (female) chains of *S. thompsoni* remain unfertilized and transform into males after 30–50 days [35] in order to fertilize subsequent female chains. This knowledge is critical for modelling approaches to more accurately incorporate the life cycle duration of salps into population dynamic and ecosystem models and needs further investigation in field studies.

Salps are known to undergo rapid individual as well as population growth, and the allocation of energy between growth and reproduction may vary over time depending on the size and reproductive status of the salps: while young individuals may invest the majority of energy into growth, energy allocation may shift from a certain timepoint onwards towards reproduction [1]. In our study, gene expression profiles in blastozooids were rather characterized by time than by fertilization status, although an interacting effect of both factors became obvious. We observed differences in energy demand, cell communication and spermatogenesis mainly between sampling timepoints, but no obvious difference between fertilized and unfertilized blastozooids could be observed. We can therefore assume that fertilization did not lead to shifts in energy allocation in our study. However, it remains unresolved if differences between timepoints would become more apparent in more advanced stages of blastozooids. Further evidence for potential physiological strategies in different developmental stages of salps is needed. In particular, the dependence of the growing embryo on the maternal blastozooid and, thus, the implications for its energy budget needs further investigation since the successful embryo development and release by its mother blastozooid is likely to be important for the persistence of a salp population [16].

## Conclusions and future directions

Understanding salp physiology and their life cycle traits is critically important to assess their ability to cope with future environmental changes such as ocean warming and associated consequences for salp habitats across the globe. Currently, the incomplete knowledge also limits our ability to accurately include and parametrize salps into models of, e.g., carbon cycling minimizing their predictive power. By creating the first transcriptomic dataset of *S. fusiformis* for gene expression analysis we could examine form-, developmental and fertilization- specific gene expression patterns. While form specific patterns were mainly characterized by an upregulation of genes involved in translation and processes related to energy provision in oozoids, the comparison within blastozooids of different developmental stages allowed first insights into closely timed processes even during the relatively short experimental duration of only a few days. We observed differences in the expression of genes involved in energy supplying processes mainly between sampling timepoints. In contrast, no obvious difference in expression magnitude between fertilized and unfertilized blastozooids was observed. In addition, using our newly generated transcriptome, several reference genes were identified and the suitability of these candidates was validated using RT-qPCR, providing a set of universal housekeeping genes and thus a basis for future process-related gene expression studies on salps. By providing these resources, we contribute to a better understanding of the processes involved in development and sexual reproduction, thereby highlighting the need for further studies on salps life cycle characteristics.

However, we acknowledge the inherent limitations of studying gene expression patterns, particularly when relying on sequence similarity to model organisms for gene ontology analysis. This approach may underestimate the true functions of genes in non-model organisms. Incorporating tissue-specific or single-cell RNA sequencing could help to reveal cell-type-specific gene expression patterns. Our study focused primarily on morphological descriptions and transcriptomic analyses of *S. fusiformis*. Although there is no documented evidence of cryptic species within *S. fusiformis*, we cannot rule out the possibility that such species exist within the sampled population. Studying cryptic biodiversity would require extensive sampling across multiple geographic regions, extensive morphological examination and detailed molecular analysis, including DNA barcoding and phylogenetics. Genetic analysis of salps is still in its early stages and can be particularly challenging. For example, Goodall-Copestake (2016) [46] reported the presence of multiple intra-individual cytochrome c oxidase subunit I (cox1, COI) barcodes in the Southern Ocean salp *S. thompsoni*, complicating species delimitation using standard genetic markers. While a comprehensive phylogenetic analysis was beyond the scope of this study, which focused on the ecological implications of the salp life cycle based on transcriptomic gene expression data, the potential presence of cryptic species should be acknowledged in future investigations—particularly in the context of biodiversity assessments. Future studies would benefit from integrating DNA barcoding with confirmatory techniques such as *in situ* hybridization to enhance species identification and validate gene expression patterns. Our study represents an important first step towards a comprehensive understanding of salp development and life cycle at both gene expression and whole animal levels, taking advantage of a unique experimental setup and integrative approach.

## Materials and methods

### Field sampling and experimental design

*Salpa fusiformis* is one of the most abundant salps in the world and is regularly observed in the bay of Villefranche-sur-Mer, France [26]. Individual sampled salps could be clearly identified on the basis of their distinct morphological characteristics. The sexual reproducing blastozooids of *S. fusiformis* sampled had an elongated body, with anterior and posterior long, narrow projections, which are very characteristic for this species. Another important morphological feature of *S. fusiformis* is the fusion of the body muscles: Muscle bands MI-MIV and MV-MVI were strongly fused dorsally. For the oozoids, there was also strong fusion of body muscles at the junctions MI-MIII and MVIII-MIX, which further distinguishes this species from others in the genus (e.g., *Salpa maxima*). Finally, during a salp bloom, we observed clear diel vertical migration (DVM) behavior in *S. fusiformis*, including a descent to deeper waters during the day, which is consistent with previous observations for this species [49,50]. For the construction of the *de novo* transcriptome of *S. fusiformis*, salps were collected during spring bloom sampling campaigns in 2017 and 2021 at the Institut de la Mer de Villefranche (France) (S1 Table in S1 File).

[1] In 2017, *S. fusiformis* was caught with a light trap at 0–5 m depth set overnight near Point B (43.685° N, 7.315667° E) at a sea surface temperature of 14 °C. Individual blastozooids and oozoids were measured for total length (TL) before stomach and embryo/stolon (if present) were removed. Samples were shock-frozen in liquid nitrogen and stored at −80 °C until further processing.
[2] In 2021, complete chains of blastozooids of *S. fusiformis* were obtained by snorkeling, using ziploc-bags and beaker in the bay of Villefranche-sur-mer. Chains were transported to the Institut de la Mer de Villefranche while keeping temperature constant during transport. After field sampling, chains of blastozooids were maintained for up to 9 days in a temperature-controlled aquarium system at *in situ* temperature of 15 °C. During the experiment, the survival rate of *S. fusiformis* in kreisel tanks was ~ 70% [23]. The aquarium system consisted of 6 small kreisel tanks in a flow-through system containing one chain of blastozooids per kreisel tank. See Müller *et al.* (2024) for a detailed maintenance protocol. For this study, individual salps from three kreisel tanks, each containing a chain of blastozooids (22–24 individuals per chain), were sampled at two timepoints, after 3 days (timepoint 1, TP1) and 7 days (timepoint 2, TP2). Individual salps were staged according to Foxton (1966), using a microscope (Nikon C-LEDS, No. 224659, China) equipped with a digital camera (Dino-Eye AM7025X, USA) followed by measurements of oral-atrial length (OAL) and total length (TL) [23]. After 7 days of maintenance, the blastozooids were still swimming actively, showed no signs of damage from contact with the walls of the kreisel tanks, and continued to develop (S1 Table in S1 File). At each sampling timepoint (TP1, TP2) one fertilized and one unfertilized blastozooid from each chain (n = 3) was sampled (Fig 3). Sampling timepoints and time window were chosen to encompass the reproductive phase of estimated ~5 days [11], when young blastozooids reach sexual maturity and are potentially fertilized (TP1), producing fully developed embryos just before they are likely to be released (TP2). Samples were shock-frozen in liquid nitrogen and stored at −80 °C until further processing at the Alfred Wegener Institute, Bremerhaven. As *S. fusiformis* is an invertebrate species that is neither endangered nor protected, no special permits were required for sampling. The number of samples collected was minimal and all specimens were handled with the utmost care.

### RNA extraction, library preparation and Illumina sequencing

Frozen tissue of salps (oozoids n = 5, blastozooids n = 5) from sampling campaign (1) was ground in a frozen state by using liquid nitrogen. RNA was extracted from each specimen using the NucleoSpin RNA Plus kit (Macherey-Nagel). About 150 mg of ground tissue was lysed in 800 μL LPB buffer containing ß-mercaptoethanol (1μl/ml LPB) using a Precellys^®^ 24 homogenizer (Bertin Technologies, France) set to 2x15 seconds (s) at 5,000 rpm at room temperature. The sample was centrifuged for 2 min at 16.000 xg at room temperature, the supernatant was transferred to the gDNA column and centrifuged again for 1 min at 11.000 xg. RNA was extracted from each specimen according to the manufacturer`s protocol with following modifications: 250 μL of Binding Buffer BS was added to the lysate. About 600 μl of whole lysate was added to the NucleoSpin^®^ RNA Plus Column and centrifuged for 1 min at 11.000 xg. The first wash step was performed with 500 μL buffer WB1 and the next two steps were performed by adding 600 μL of buffer WB2. The column was dry centrifuged at 11.000 xg. Total RNA was eluted with 30 μL nuclease-free water and centrifuged at 11.000 xg for 2 min, and re-eluted. The extraction procedure was performed in duplicates for each blastozooid sample and the elutes were then pooled and reprecipitated with 0.1 volumes of 3M sodium acetate pH 5.2 and 2.5 volumes of ice-cold 100% ethanol. RNA was incubated overnight at −20 °C and then centrifuged at 20.000 xg at 4 °C for 45 min. The pellet was washed twice with 500 μL ice cold 75% ethanol and centrifuged for 10 min at 20.000 x g and 4°C after each washing step. A short centrifuge step was added at the end to remove any remaining ethanol. After air drying the pellet for 5 min, RNA was resuspended in 35 μL nuclease free water and stored at −80 °C.

Frozen salps (blastozooids n = 12) from sampling campaign (2) were transferred to RNAlater^®^ ICE (Thermo Fisher Scientific, USA) for 24 h at −20 °C prior to the extraction of total RNA. The stomach and embryo, if present, were dissected and the remaining tissue was transferred to 3 ml of TRI^®^ Reagent supplied by the Direct-zol RNA MiniPrep Kit (R2051, Zymo Research). Tissue was homogenized using a Precellys^®^ 24 homogenizer (Bertin Technologies, France) set to 2x15 seconds (s) at 5,000 rpm at room temperature. Chloroform (Sigma-Aldrich, USA, 600 μL, Factor 1:5 Chloroform: TRI^®^ Reagent) was added, vortexed, incubated for 2 min and centrifuged for 15 min at 12.000 xg and 4 °C. RNA was further purified using the standard protocol for the Direct-zol RNA MiniPrep kit (R2051, Zymo Research) using Zymo-Spin IIC Columns (C1011, Zymo Research). Total RNA was eluted with 30 μL nuclease-free water.

Total RNA of all samples was quantified by NanoDropTM 2000 (Thermo Fisher Scientific, Waltham, MA, USA). RNA integrity of each sample was assessed by capillary electrophoresis using the RNA 6000 Nano LabChip and the Agilent Bioanalyzer 2100 (Agilent Technologies, Santa Clara, CA, USA). Only high-quality RNA samples (RIN = 9–10) were used for library construction (S1 Table in S1 File). RNA-Sequencing of each individual sample was carried out from IGA Technology Services (Udine, Italy). cDNA libraries were constructed with 500 ng of total RNA by using the TruSeq Stranded mRNA Sample Prep kit (Illumina, San Diego, CA, USA) following the manufacturer’s instructions. Total RNA samples and final cDNA libraries were quantified with the Qubit 2.0 Fluorometer (Invitrogen, Carlsbad, CA, USA) and quality tested by Agilent 2100 Bioanalyzer Nano assay. For cluster generation on the flow cell, libraries were processed with cBot (Illumina, San Diego, CA, USA). Sequencing was carried out on paired-end mode by using HiSeq 2500 (Illumina) with a targeted sequencing depth of about 80 million reads per sample (125 bp) for specimens obtained during sampling campaigns (1) and by using NovaSeq6000 of about 80 million reads per sample (150 bp) for individuals of sampling campaign (2). Raw sequencing data are accessible within NCBI`s Sequence Read Archive (SRA) database under Bioproject Accession Number PRJNA995943.

### *De novo* transcriptome assembly, quality assessment and annotation

Illumina short reads were first processed using Cutadapt software [51] (*v.* 2.7) to remove adapter sequences and other types of undesired sequences. The quality of trimmed reads was checked with the program FastQC [52]. Trimmed reads were further processed through digital normalization, following the khmer protocol [53]. This additional step was performed with the aim of removing redundant and poor-quality reads from the input dataset. First, paired reads were interleaved to combine forward and reverse reads into a single file (using the khmer script “interleave-reads.py”). Digital normalization was performed using the khmer script “normalize-by-median.py”, removing reads with a median k-mer abundance higher than the desired coverage (k-mer selected size = 20, cutoff = 20). Additionally, a filter step was applied to remove low k-mer abundance using the khmer script “filter-abund.py”. After normalization, the khmer script “extract-paired-reads.py” was used to split the reads into pairs and orphans.

Based on this high-quality read subset, five different *de novo* assemblers were used to reconstruct the transcriptome using the workflow proposed by Urso *et al.* (2022) [54], which has also been used previously for another salp species, *Salpa thompsoni* [29]: Trinity *v.* 2.13.2 [55], BinPacker *v.* 1.1 [56], IDBA-tran *v*. 1.1.3 [57], rnaSPAdes *v*. 3.14.1 [58] and TransABySS *v*. 2.0.1 [59]. This approach aimed to maximize the possibility of generating the most reliable transcriptome reference, to overcome the limitations associated with each specific assembler, and to reduce the number of artefacts and redundancies that are typical problems associated with *de novo* transcriptome reconstructions. The resulting transcriptomes were then processed through a series of filtering steps to minimize the presence of artefacts and redundancies. Transcript quantification analysis was performed using the Salmon software *v.* 1.3.0 [60].

All transcripts with more than ten mapped reads per samples were retained. The results of all assemblers were merged and the “cd-hit-est” program was run (Li and Godzik, 2006; *v.* 4.8.1) [61] with the following parameters: -c 0.95-M 100000-T 22. All sequences that shared ≥ 95% of their content were collapsed using this setup. To select the best reconstruction of each assembler, the EvidentialGene tr2aacds pipeline (Gilbert, 2013; *v*. 4) [62] was applied. Specifically, the “tr2aacds” tool was used with the following parameter settings (-NCPU = 22 -MAXMEM = 100000 -logfile -tidyup -species = *Salpa fusiformis*) to select the best reconstruction for a given transcript among all assemblies. In order to identify redundant or incorrectly assembled sequences that were still present in the transcriptome, all transcript sequences were cross-checked with the blastn tool. All sequences already over 90% represented in a longer transcript were removed. A second round of abundance quantification was performed using Salmon *v.* 1.3.0 [60], removing all transcripts with an average abundance below 0.1 TPM. Quality assessment of the assemblies was obtained using the Trinity script *“Trinity_stats.pl”*, computing basic statistics including the number of total transcripts, percent of guanine-cytosine (GC) content, the average fragment length, the total number of bases and the N50 index. In addition, the transcriptome completeness was assessed using BUSCO v. 4.1.4 [63], calculating the number of complete (single-copy and duplicated), fragmented and missing genes.

Following the suggestion of Soneson et al. (2016), the number of transcripts was pooled at the gene-level to increase the accuracy, power and interpretability of downstream analyses [64]. Functional annotation was performed aligning the assembled transcripts against the NCBI NR (non-redundant) UniProtKB/TrEMBL protein databases and against the NCBI NT nucleotide collection (download date 22/04/2021). A InterproScan v. 5.51–85.0 [65] analysis was run to search for known functional domains and to predict protein family membership.

### Transcriptome analysis, differential gene expression and gene ontology enrichment analysis

For the analysis of the transcriptome GO term distribution, R package GO.db *v.* 3.13.0 [66] was used, which allows the identification of ancestors for each level of the term of interest. Samples from both campaigns (1 and 2, S1 Table in S1 File) were used for differential gene expression and gene ontology enrichment analysis. Normalization was conducted separately for each campaign using Bioconductor R package DESeq2 *v.* 1.32.0 [67]. Variation within and between groups was estimated from principal component analysis (PCA) based on variance-stabilized normalized read counts generated via the *vst* function in DESeq2. For the comparison between reproductive forms, gene expression analysis was performed between blastozooids and oozoids from campaign 1. In order to analyze the differences between the two sampling timepoints (TP1, TP2) in relation to the fertilization status of blastozooids, samples from the kreisel tank system (campaign 2) were used. The following comparisons were examined:

(a)  Analysis of differences in gene expression between fertilization status (fertilized vs. unfertilized) at TP1 and TP2
(b)  Analysis of differences in gene expression between timepoints (TP1 *vs.* TP2) within fertilized blastozooids
(c)  Analysis of differences in gene expression between timepoints (TP1 *vs.*TP2) within unfertilized blastozooids
(d)  Analysis of shared genes, differentially expressed genes over time (TP1 *vs.* TP2) in both, fertilized (b) and unfertilized blastozooids (c)
(e)  Analysis of the main effect of time (TP1 *vs.* TP2) on differential gene expression, independent of fertilization status (after subtracting interaction genes identified in (b) and (c), respectively)

Only genes with Benjamini-Hochberg adjusted p- value < 0.05 and a onefold difference (by setting the log fold change threshold (LFCT) to 0) in expression levels were accepted as significantly differentially expressed. For the gene ontology (GO) term enrichment analysis of biological processes (BP), both up- and downregulated genes were considered using the *weight01* algorithm implemented in topGO *v.* 2.44.0 [68]. The *Gene Universe* for this analysis consisted of the full set of annotated genes from our RNA-seq dataset. Only GO terms with a p-value < 0.05 (Fishers exact test) were considered significant. The transcriptome annotations were additionally filtered for keywords related to spermatogenesis (spermatogenesis-associated protein|sperm-associated protein) to detect genes involved in protogynous hermaphroditic differentiation in the sexual reproductive life history.

### Real-Time quantitative PCR validation

For RT-qPCR validation of RNA-seq results, five representative target genes (*atpsyn, auto, cdan, sperm48 and tyramino*) were chosen from lists generated by DGE and GO term enrichment analysis. Genes with a minimum mean expression of 300 and greatest log2 fold change were selected. To our best knowledge, no RT-qPCR analysis has been performed for salps to date, and information on appropriate reference genes is lacking. Therefore, we aimed to identify and validate a set of reference genes with stable expression under different conditions, ensuring robust validation of RNA-seq results and providing essential molecular tools for future studies. We identified potential reference genes that showed the greatest stability in the transcriptome across all forms and stages by using a combination of conventional literature screening (approach 1) and expression stability analysis (approach 2). From both approaches, three potential reference candidates (*ef1, act* and *ubq* (approach 1)*, fip1, pp2a* and *snare* (approach 2)) were selected for evaluation by RT-qPCR. Additional details of the selection of reference genes are provided in the supplement (S1 Text, S9–S11 Tables in S1 File). Transcripts of target and reference genes for primer design were selected according to greatest alignment of transcript and score. Primer pairs were designed by Primer 3 software [69]. After design, the primers were checked for specificity via primer Blast (NCBI) and the generated transcriptome in this study. The information on all tested genes is listed in S12 Table in S1 File.

First-strand cDNA synthesis of all samples was performed using the High-Capacity Kit (Applied Biosystems, CA, USA) starting from 100 ng of total RNA in a final volume of 20 μL according to the manufacturer’s instructions. RT-qPCRs were performed in duplicate using Viia 7 system (Applied Biosystems, USA) and PowerUP SYBR green master mix chemistry (Thermo Fisher Scientific, USA). Amplifications were carried out in in a total volume of 10 μL containing 5 μL PowerUP SYBR green master mix, 3 μL cDNA (1.5 ng cDNA), 0.6 μL (0.3 μM) of each specific primer and 0.8 μL water. The qPCR cycling conditions were 95°C for 10 min, 40 cycles (95°C for 15 s and 60°C for 1 min), and a final step at 95 °C for 15 s, 60 °C for 1 min, and 95 °C for 15 s. The PCR efficiency of each gene was calculated from the slope given after running standard curves generated with 5 points of serial dilutions of cDNA (3ng- 0.075ng) using the following formula: E = 10^(−1/slope)^, where E = 2 and corresponds to 100% efficiency. Expression data were obtained as quantification cycle (Cq) values. Efficiency corrected Cq values were calculated for each sample and gene by the following equation:


$$CqE=Cq*\left(\frac{\log(E)}{\log(2)}\right)$$

The stability of the six candidate reference genes was determined by geNorm using the R package *ctrlGene v*. 1.0.1 [70]. The geNorm algorithm calculates an average expression stability value (M) for each gene [71]. The M value is defined as the average pairwise variation in a given gene compared to other given potential reference genes. Genes with the lowest M tend to be the most stable expressed. The minimum number of genes required for normalization can be determined by pairwise variation V_n_/V_n+1_ [71]. In order to compare the results obtained by geNorm via R package *ctrlGene*, the efficiency-corrected Cq values were processed using the web-based analysis tool RefFinder (http://blooge.cn/RefFinder/) that integrates geNorm [71], Normfinder [72], BestKeeper [73] and comparative Δ Cq method [74]. Based on the rankings generated by each algorithm, a weight is assigned to each gene and geometric means of the gene weights are calculated for a comprehensive final ranking. In our study, the M value of all potential reference candidates was < 0.5 which is the threshold for appropriate reference gene selection, confirming the suitability of all candidates (S1 Fig, S13, S14 Tables in S1 File). The reference genes *ubq* and *snare* were used for normalization as this combination showed the highest stability (M = 0.25) for all samples. Additional details on the results of the stability analysis are provided in the supplemental text (S1 Text). The fold change (2^-ΔΔCt^) was calculated using oozoids and TP2 as control, for the comparison between reproductive forms and developmental stages, respectively.

### Visualization

Data processing and visualization was done with help of *tidyverse v.* 1.3.2 [75].

## Supporting information

S1 Fig Analysis of gene expression stability across three different sets of samples/conditions. Sample sets reflect the conditions of all samples (n = 21), of different forms (blastozooids and oozoids, (n = 9, campaign 1)) and of different states of fertilization at different sampling timepoints (n = 12, campaign 2). (A) Raw Cq values, corrected for primer efficiency of 6 reference gene candidates as well as the arithmetic mean ± standard deviation for each gene is given. Point labels depict the coefficient of variance (CV). (B) Expression stability values (M) were calculated using geNorm for each reference gene candidate/combination. The dashed line indicates the M = 0.5 value, which is the threshold for appropriate reference gene selection. (C) The optimal number of reference genes required for normalization was determined by calculating pairwise variation values (V_n_/V_n+1_). Generally, a variation value < 0.15 indicates the minimum number of genes recommended for normalization.
(PDF)

S1 Text Selection and stability analysis of reference gene candidates for RT-qPCR of *S. fusiformis.* (PDF)

S1 File **S1 Table**. Overview of samples used for the de novo transcriptome of *S. fusiformis*. RNA concentration and quality of each sample was verified using NanoDropTM 2000 (Thermo Fisher Scientific, USA). RIN- a measure of integrity was measured by Bioanalyzer 2100 (Agilent Technologies, USA). **S2 Table**. Results of GO term enrichment analysis by topGO combined with results of DGE analysis by DESeq2: form comparison (campaign 1). **S3 Table**. Results of GO term enrichment analysis by topGO combined with results of DGE analysis by DESeq2: fertilized vs not fertilized (at TP1) (campaign 2). **S4 Table**. Results of GO term enrichment analysis by topGO combined with results of DGE analysis by DESeq2: fertilized vs not fertilized (at TP2) (campaign 2). **S5 Table**. Results of GO term enrichment analysis by topGO combined with results of DGE analysis by DESeq2: TP1 vs TP2 (fertilized) (campaign 2). **S6 Table**. Results of GO term enrichment analysis by topGO combined with results of DGE analysis by DESeq2: TP1 vs TP2 (unfertilized) (campaign 2). **S7 Table**. Results of GO term enrichment analysis by topGO combined with results of of DGE analysis by DESeq2: shared DEG of fertilized and unfertilized blastozooids (campaign 2). **S8 Table**. Results of GO term enrichment analysis by topGO combined with results of DGE analysis by DESeq2: main effect time (campaign 2). **S9 Table**. List of potential reference genes candidates used in ascidian studies and their mean expression, standard deviation and rank in the generated CV ranked list of S. fusiformis (approach 1). **S10 Table**. List of potential reference genes candidates detected in CV ranked list of S. fusiformis with <0.3 log fold change (approach 2). **S11 Table**. Raw Cq values of reference gene candidates after efficiency correction. **S12 Table**. Information of the candidate reference and target genes, including primer sequences, size, GC (%), melting temperature of primers and size of amplicons. Efficiency was calculated by E = 10(−1/slope). **S13 Table**. Expression stability values. **S14 Table**. Stability values and ranking of 6 reference gene candidates.
(XLSX)
