# Supplementary material for: Time matters: Transcriptomic insights into temporally regulated reproductive and physiological processes in the life cycle of salps
Source: PLoS One. 2025 Jun 20;20(6):e0326246. doi: 10.1371/journal.pone.0326246 (PMC12180652; doi:10.1371/journal.pone.0326246)
Supplement: S1 Fig — Sample sets reflect the conditions of all samples (n = 21), of different forms (blastozooids and oozoids, (n = 9, campaign 1)) and of different states of fertilization at different sampling timepoints (n = 12, campaign 2). (A) Raw Cq values, corrected for primer efficiency of 6 reference gene candidates as well as the arithmetic mean ± standard deviation for each gene is given. Point labels depict the coefficient of variance (CV). (B) Expression stability values (M) were calculated using geNorm for each reference gene candidate/combination. The dashed line indicates the M = 0.5 value, which is the threshold for appropriate reference gene selection. (C) The optimal number of reference genes required for normalization was determined by calculating pairwise variation values (Vn/Vn+1). Generally, a variation value < 0.15 indicates the minimum number of genes recommended for normalization. (PDF) [file pone.0326246.s001.pdf]

Supplementary Figures

Time matters: Transcriptomic insights into temporally regulated reproductive and physiological processes in the life cycle of salps

Svenja J. Müller, Ilenia Urso, Sara Driscoll, Katharina Michael, Gabriele Sales, Cristiano de Pittà, Wiebke Wessels, Bettina Meyer

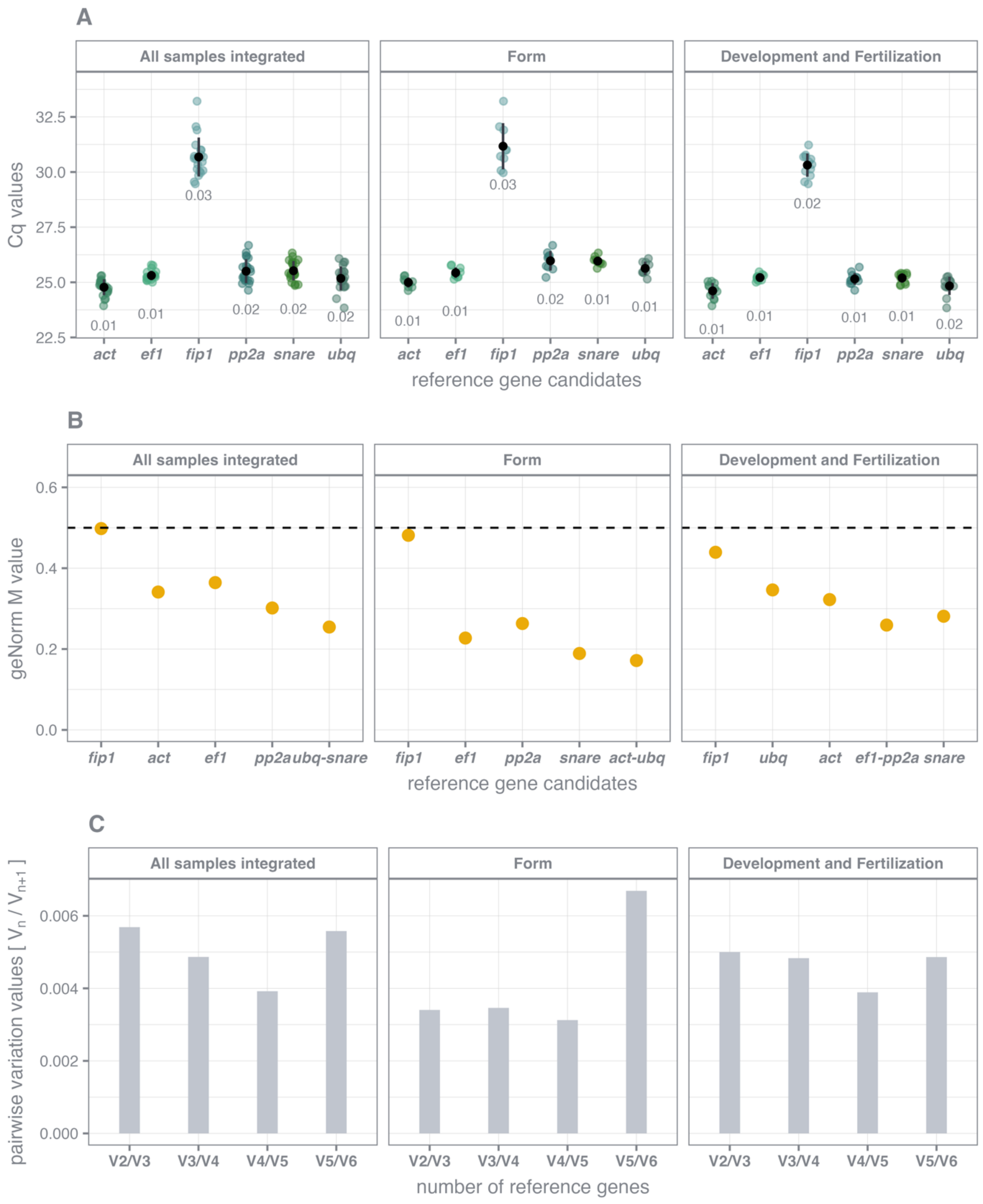

Figure S1. Analysis of gene expression stability across three different sets of samples/conditions. Sample sets reflect the conditions of all samples (n=21), of different forms (blastozooids and oozoids, (n=9, campaign 1)) and of different states of fertilization at different sampling time points (n=12, campaign 2). (A) Raw Cq values, corrected for primer efficiency of 6 reference gene candidates as well as the arithmetic mean ± standard deviation for each gene is given. Point labels depict the coefficient of variance (CV). (B) Expression stability values (M) were calculated using geNorm for each reference gene candidate/combination. The dashed line indicates the M=0.5 value, which is the threshold for appropriate reference gene selection. (C) The optimal number of reference genes required for normalization was determined by calculating pairwise variation values (V_n/V_{n+1}). Generally, a variation value < 0.15 indicates the minimum number of genes recommended for normalization.
