# Supplementary material for: Time matters: Transcriptomic insights into temporally regulated reproductive and physiological processes in the life cycle of salps
Source: PLoS One. 2025 Jun 20;20(6):e0326246. doi: 10.1371/journal.pone.0326246 (PMC12180652; doi:10.1371/journal.pone.0326246)
Supplement: S1 Text — (PDF) [file pone.0326246.s002.pdf]

Supplementary Text

Time matters: Transcriptomic insights into temporally regulated reproductive and physiological processes in the life cycle of salps

Svenja J. Müller, Ilenia Urso, Sara Driscoll, Katharina Michael, Gabriele Sales, Cristiano de Pittà, Wiebke Wessels, Bettina Meyer

Text S1. Selection and stability analysis of reference gene candidates for RT-qPCR of *S. fusiformis*

Methods: Selection of reference gene candidates

To detect suitable reference genes with the greatest stability under different conditions, we used a combination of expression stability analysis and conventional literature screening. First, expression stability of all samples was examined by calculating the coefficient of variance (CV) according to the following equation:

$$CV = \frac{Standard\ Deviation}{Mean\ Expression}$$

A filter for mean expression (mean > 300 & mean < 6000) and CV < 0.3 was applied to identify genes with comparatively high expression that are robustly expressed between conditions. Based on this CV ranked list, we applied a dual approach in order to test expression stability of (1) traditional reference candidate genes (2) potential reference genes with lowest differential gene expression.

(1) In a first approach, traditional reference genes (n=14) previously used in ascidian studies (1–4) were searched in the CV ranked list.

(2) In a second approach, the fold change between conditions was taken into consideration in order to avoid the selection of reference candidate genes affected by specific conditions. Therefore, genes with a high fold change > 0.3 log fold change (LFC) for pairwise comparisons detected by Bioconductor R package DESeq2 *v.* 1.32.0 (5) were eliminated in the CV ranked list. Only genes with annotation were considered.

From both approaches, three potential reference candidates were selected for evaluation by RT-qPCR.

Results: Selection and stability analysis of qPCR reference gene candidates for molecular studies on *S. fusiformis*

In order to identify potential reference genes with comparatively high and stable expression levels under different conditions, we applied a mean expression and variance of coefficient (CV) filter (mean > 300 & mean < 6,000, CV < 0.3) to all samples, resulting in a final ranked list of 1,320 potential reference genes. Only 4 out of 14 genes met the stability criteria (CV < 0.3) when examining "traditional" reference genes (approach 1, Table S9). While *elongation factor 1*, *actin*, *ubiquitin-conjugating enzyme E2* and *cyclophilin* were most stable, all four genes encoding for ribosomal proteins (*rpl17, rps15, rpl11, rps27A*) showed considerable variations across our samples. By applying a logfold change filter to the CV ranked list (LFC < 0.3, approach 2) a list of 12 potential candidates was obtained (Table S10). The three genes *ef1* (*Elongation factor 1*), *act* (*Actin-related protein 3*) and *ubq* (*Ubiquitin-conjugating enzyme E2*) from the first approach and *fip1* (*Pre-mRNA polyadenylation factor Fip1 domain*), *pp2a* (*Serine/Threonine protein phosphatase 2A, regulatory B subunit, B56)* and *snare* (*t-snare protein*) from the second approach were selected for further evaluation of expression stability via RT-qPCR (Table S12). In our analysis, all reference candidate genes had a linear regression coefficient (R2) > 0.997 and amplification efficiency of 1.95-2.06.

The expression levels of all six reference gene candidates were verified in three different sets of samples to test their suitability for different conditions: (set 1) all samples integrated (n=21), (set 2) samples of different form (blastozooids and oozoids from campaign 1, n=9) and (set 3) samples of different states of fertilization and sampling timepoints (from campaign 2, n=12). Cq values of all candidate genes ranged from 23.84 to 33.21 (Figure S1A, Table S11). Across all sets, candidate genes showed similar Cq value ranges. The gene with highest Cq variation (standard deviation, SD) was *fip1* in all sets (± SD ranged from 0.53 and 1.05) with a CV of 0.02-0.03, while *snare*, *act* and *ef1a* showed lowest variability (± SD ranged from 0.13-0.44) and a CV of 0.01-0.02.

In addition, their stability and suitability were tested using geNorm, implemented in the R package *ctrlGene v.* 1.0.1, which calculates the expression stability value (M) for each reference candidate gene and the pairwise variation (V) value to determine the minimum number of genes required for accurate normalization. M ≤ 0.5 has been suggested as a threshold for appropriate reference gene selection and 0.15 is a suitable cut-off value in pairwise variation

analysis (6,7). In our study, all M values were lower than 0.5 for all different sets tested, and V-value of V2/3 was below the default threshold (Figure S1B, C). Based on the results of geNorm most suitable reference gene combinations for all samples integrated (set 1) was *ubq-snare* (M= 0.25), for samples covering different forms (set 2) *act-ubq* (M= 0.17) and for samples of different fertilization and development stages (set 3) *ef1a-pp2a* (M= 0.26) (Table S13). To validate the results obtained by geNorm and to compare them with other methods, we used the RefFinder tool, which combines the results of four methods into a comprehensive ranking based on the stability of the reference gene candidates. By using the web-based tool RefFinder, the same results were obtained as with geNorm using the *ctrlGene* package (Table S14). Depending on the set and method used, *snare*, *pp2a*, *ef1* and *ubq* were the most stable, while *fip1* was always the least stable gene, confirming the results of geNorm.

Discussion: Suitability of reference genes selected

The accuracy of real-time quantitative (RT-qPCR) depends on the accuracy of normalization using appropriate reference genes to reduce variation and technical noise. To our best knowledge, no RT-qPCR analysis has been performed for salps so far. To test the "power" of our transcriptome, not only to detect gene expression patterns as described above, but also to serve as a database to provide useful molecular tools for future studies, we aimed to identify and validate a set of reference genes that are stably expressed under different conditions (here: field/kreisel tank, form, fertilization and developmental stages).

During the literature- based approach (1), the screening of the generated CV ranked list for genes commonly used as reference genes in different ascidian species (*Ciona savignyi, Ciona intestinalis, Botryllus schlosseri*) resulted in only 4 (*elongation factor 1*, *actin*, *ubiquitin-conjugating enzyme E2* and *cyclophilin*) out of 14 genes (Table S9). In the second approach, potential new reference genes with the lowest differential gene expression in the CV ranking were sought by applying a log fold change filter (LFC < 0.3) to avoid selecting reference gene candidates affected by specific conditions, resulting in 12 potential new reference gene candidates (Table S10). The generally low number of reference genes found here in both approaches (4 and 12, respectively) illustrates the difficulty in obtaining suitable candidate reference genes, especially with respect to various different experimental and biological conditions as in our study (field/kreisel tank, form, fertilization and developmental stages). However, it should be noted that only genes with correct annotations were selected, so the number of suitable candidates was additionally reduced by neglecting possible genes without annotations.

No genes encoding for ribosomal proteins were found to be suitable in our study, which is in contrast to the study by Huang *et al.* (2016) and Stewart-Clark *et al.* (2013), who used ribosomal proteins as reference genes in *Ciona savignyi* and *Ciona intestinalis*, respectively. This is also in contrast to studies, that used ribosomal proteins (e.g., 18srna, rps13) for normalization of gene expression in krill (8,9) and fish (10,11). In salps, genes encoding ribosomal differences were found to be the main difference between the two forms (blastozooids and oozoids) in *S. thompsoni* during winter (Müller *et al.* 2022), but also in *S. fusiformis* in spring in the present study. Our results therefore suggest that, at least for salps, ribosomal proteins may not be suitable as reference genes when both forms of salps (blastozooids and oozoids) are considered, as was the case in our study.

We evaluated the stability of three genes of the first (*ef1, act, ubq*) and the second approach (*fip1, pp2a, snare*) in all three sets of samples using RT-qPCR (Figure S1). Our analysis showed that both reference gene selection approaches yielded candidate genes which displayed stable gene expression patterns across all sets of conditions, as validated by applying the thresholds of geNorm and the RefFinder tool (Figure S1B, C). *fip1* was the least stable candidate gene for all sets of conditions tested and is therefore likely the least suitable candidate among our gene set. Based on the fact that normalization by multiple housekeeping genes instead of only one is recommended, and geNorm has to been shown to be most affected by efficiency, we decided to follow the suggestion by geNorm and normalized the Cq values of 5 target genes by a combination of *ubq* and *snare* to validate gene expression differences detected in DEG analysis (6). Results by RT-qPCR not only showed that the expression trends were similar between qPCR and RNA-seq and therefore, proves the accuracy of our findings on differential gene expression discussed here (Figure 6), but also confirms accurate normalization by the selection of our reference genes.

This is the first study to validate reference genes for RT-qPCR analysis in salps and also the first study to use RNA sequencing data for reference gene selection in tunicates in general. Our transcriptome as well as the successful validation of the reference gene set selected therefore provides a new resource for upcoming molecular studies in more detail on salps. Furthermore, we show that RNA-seq is a promising tool to not only validate commonly used reference genes before performing RT-qPCR, but also to discover novel genes through a series of filtering steps, which is a very promising and powerful tool, especially when studying non-model organisms.

References

1. Huang X, Gao Y, Jiang B, Zhou Z, Zhan A. Reference gene selection for quantitative gene expression studies during biological invasions: A test on multiple genes and tissues in a model ascidian Ciona savignyi. Gene [Internet]. 2016;576(1):79–87. Available from: http://dx.doi.org/10.1016/j.gene.2015.09.066
2. Rodriguez D, Sanders EN, Farell K, Langenbacher AD, Taketa DA, Hopper MR, et al. Analysis of the basal chordate Botryllus schlosseri reveals a set of genes associated with fertility. BMC Genomics. 2014;15(1).
3. Stewart-Clark SE, Davidson J, Greenwood SJ. Monitoring for propagules of Ciona intestinalis in marine water samples: The development of temporal gene expression markers for viability and life stage specific assays. Manag Biol Invasions. 2013;4(3):207–17.
4. Campagna D, Gasparini F, Franchi N, Vitulo N, Ballin F, Manni L, et al. Transcriptome dynamics in the asexual cycle of the chordate Botryllus schlosseri. BMC Genomics [Internet]. 2016;17(1):1–17. Available from: http://dx.doi.org/10.1186/s12864-016-2598-1
5. Love MI, Huber W, Anders S. Moderated estimation of fold change and dispersion for RNA-seq data with DESeq2. Genome Biol. 2014;15(12):1–21.
6. Vandesompele J, De Preter K, Pattyn F., Poppe B, Van Roy N, De Paepe A, et al. Accurate normalization of real-time quantitative RT-PCR data by geometric averaging of multiple internal control genes. Genome Biol. 2002;3.
7. Hellemans J, Mortier G, De Paepe A, Speleman F, Vandesompele J. qBase relative quantification framework and software for management and automated analysis of real-time quantitative PCR data. Genome Biol. 2007;8(2).
8. De Pittà C, Bertolucci C, Mazzotta GM, Bernante F, Rizzo G, De Nardi B, et al. Systematic sequencing of mRNA from the Antarctic krill (Euphausia superba) and first tissue specific transcriptional signature. BMC Genomics [Internet]. 2008 Dec 28;9(1):45. Available from: https://bmcgenomics.biomedcentral.com/articles/10.1186/1471-2164-9-45
9. Piccolin F, Meyer B, Biscontin A, Pittà C De, Kawaguchi S, Teschke M. Photoperiodic modulation of circadian functions in Antarctic. 2018;38(September):707–15.
10. Rojas-Hernandez N, Véliz D, Vega-Retter C. Selection of suitable reference genes for gene expression analysis in gills and liver of fish under field pollution conditions. Sci Rep [Internet]. 2019 Mar 5;9(1):3459. Available from: https://www.nature.com/articles/s41598-019-40196-3
11. Olsvik PA, Søfteland L, Lie KK. Selection of reference genes for qRT-PCR examination of wild populations of Atlantic cod Gadus morhua. BMC Res Notes [Internet]. 2008;1(1):47. Available from: http://bmcresnotes.biomedcentral.com/articles/10.1186/1756-0500-1-47
12. Müller SJ, Michael K, Urso I, Sales G, De Pittà C, Suberg L, et al. Seasonal and Form-Specific Gene Expression Signatures Uncover Different Generational Strategies of the Pelagic Tunicate Salpa thompsoni During the Southern Ocean Winter. Front Mar Sci [Internet]. 2022 Jun 16;9(June). Available from: https://www.frontiersin.org/articles/10.3389/fmars.2022.914095/full
